# The effect of coaching on the simulated malingering of memory impairment

**DOI:** 10.1186/1471-2377-8-37

**Published:** 2008-10-07

**Authors:** Jascha Rüsseler, Alexandra Brett, Ulrike Klaue, Michael Sailer, Thomas F Münte

**Affiliations:** 1 Department of Psychology II, Neuropsychology Unit, Otto-von-Guericke University Magdeburg, Germany; 2 Center for Behavioral Brain Sciences, Magdeburg, Germany; 3 Median Klinik NRZ, Magdeburg, Germany; 4 Klinik für Neurologie II, Otto-von-Guericke University Magdeburg, Germany; 5 Institut für Psychologie II, Abt. Neuropsychologie, Postfach 4120, 39016 Magdeburg, Germany

## Abstract

**Background:**

Detecting malingering or exaggeration of impairments in brain function after traumatic brain injury is of increasing importance in neuropsychological assessment. Lawyers involved in brain injury litigation cases routinely coach their clients how to approach neuropsychological testing to their advantage. Thus, it is important to know how robust assessment methods are with respect to symptom malingering or exaggeration.

**Methods:**

The influence of different coaching methods on the simulated malingering of memory impairments is investigated in neurologically healthy participants using the Short-Term-Memory Test from the Bremer Symptom-Validierung (STM-BSV). Cut-offs were derived from patients with mild to severe traumatic brain injury. For comparison purposes, the German adaptation of the Rey Auditory Verbal Learning Test (AVLT), and the Rey 15 Items Test (FIT) were additionally administered. Four groups of neurologically healthy subjects were instructed to (1) perform as best as they can, (2) simulate brain injury, (3) simulate brain injury and received additional information about the sequelae of head trauma, (4) simulate brain injury and received additional information on how to avoid detection. Furthermore, a group of patients with mild to severe closed head injury performed the tests with best effort.

**Results:**

The naïve simulator and the symptom coached groups were the easiest to detect, whereas the symptom plus test coached group was the hardest to detect. The AVLT and the FIT were not suited to detect simulators (sensitivities from 0% to 50.8% at 75% specificity) whereas the STM-BSV detected simulators with 67% – 88% sensitivity at a specificity of 73%. However, the STM-BSV was not robust to coaching.

**Conclusion:**

The present investigation shows that symptom validity testing as implemented in the BSV-STM is one clinically useful element in the detection of memory malingering. However, clinicians have to be aware that coaching influences performance in the test.

## Background

The detection of malingering or exaggeration of impairments in brain function is of increasing importance in clinical neuropsychological assessment. In a forensic setting, an estimated 70% or more of patients assessed by clinical neuropsychologists are suspected to alter their presentations [1,2]. Memory impairment is one common symptom of brain injury that is well-known to laypersons. For example, 82% of the general public are aware that a concussion often results in memory problems [3]. Individuals who attempt to malinger head trauma symptoms often report a variety of memory difficulties [4] and perform poorly on memory tests [5]. Patients with brain injury also often complain of difficulties to remember things [6], and their performance on memory tests is impaired [7].

It is considered standard practice for neuropsychologists in North America to use measures for malingering detection routinely [8-10]. In contrast, effort testing has had limited impact on clinical practice in European countries. Notable exceptions are the Amsterdam Short-Term Memory Test [11], adaptations of Green's Word Memory Test [12] to several European languages, and the "Testbatterie zur Forensischen Neuropsychologie" (TBFN; [13]). The TBFN contains 23 tests specifically designed to detect malingering (a computerized version of Rey's 15 Item Test, FIT [14]; an auditory analog version of Rey's 15 Item Test; two tests for the assessment of memory in everyday life; the Bremer Symptom-Validierung, BSV: 19 symptom validity tests to assess perceptual and mnestic impairments). The present study uses an analog design to evaluate the usefulness of the BSV short-term memory assessment subtest, the FIT and the VLMT (Verbaler Lern- und Merkfähigkeitstest [15], German adaptation of Rey's Auditory Verbal Learning Test) to detect malingering of memory impairment. Furthermore, the effects of different coaching procedures on classification rates are investigated.

### Approaches for the detection of memory malingering

Three basic approaches for malingering detection have been proposed: looking for inconsistencies in test results [16], the use of tests specifically designed to detect incomplete effort, and the application of cut-off values derived from standard neuropsychological tests. The most-widely used groups of tests specifically designed to detect incomplete effort are (a) tests that appear to be more difficult than is actually the case (e.g., FIT), and (b) the symptom validity technique.

#### Tests appearing more difficult than they actually are

The FIT is introduced as a very difficult memory test as it requires to remember 15 different items in a short time. In fact, the test is very simple because of the redundancy among the items, and patients with significant brain impairment can perform the test without much difficulty. The rationale of the test assumes that malingerers are unaware of this fact and reason that, in order to be categorized as memory impaired, they will have to recall only a few items. Thus, patients with brain impairment will do well on the FIT, whereas malingerers perform poorly and can thus be identified [17-19].

#### Symptom validity testing

In the symptom validity technique, each item has a 50% probability of obtaining a correct response when guessing. Theoretically, a person scoring below chance is most likely malingering. Prominent examples of this technique are the Test of Memory Malingering (TOMM; [20]), and the Portland Digit Recognition Test, [21]).

Symptom validity tests require that participants believe that they have to perform a difficult task. If the malingerer does not realize that the task is easy, he will perform poorly. However, if the patient notices that the task is easy, he might recognize the attempt to detect malingering and, thus, perform normally on the task. In this context, it is interesting that 48% of US lawyers believe that they should provide information about psychological tests to their clients [22], and that lawyers involved in brain injury litigation cases indeed do this regularly [23]. Furthermore, the internet provides an easily accessible source of information about tests of malingering detection that can be used by patients to prepare themselves for a neuropsychological assessment [24]. Thus, litigants may well be aware of the rationale of symptom validity testing.

#### Standard memory tests

One approach to overcome these criticisms of the symptom validity tests is the use of measures derived from standard neuropsychological procedures. Studies on the usefulness of cut-offs derived from standard neuropsychological tests have yielded mixed results. For example, the Rey Auditory Verbal Learning Test (AVLT) has been used to detect poor effort in personal injury litigants. Various measures derived from the test have been studied. Classification rates ranged from 13% to 76% at specificities of 90% or above. Note that only two studies report one measure each with a sensitivity above 70% (data taken from [25]). It seems that bona fide patients do perform poorly on this test of memory function making the discrimination between real and malingered deficits difficult. However, Barrash et al. [26] proposed an extended version of the AVLT (additional recognition trial after 60 minutes) that yielded better results. We included the German version of the AVLT (i.e., the VLMT) in our assessment as it is a commonly used memory test that can be administered in a reasonable amount of time.

Other measures derived from a variety of standard neuropsychological tests yielded more promising results (for a recent review, see [26]).

### The effects of coaching on the detection of memory malingering

As stated above, coaching affects the validity of tests of memory malingering. Thus, it is important to study effects of coaching on different single or combined measures used to detect feigned memory performance. In previous research employing analog designs, healthy participants were instructed to feign memory impairment and were provided with different amounts of information on the sequelae of brain injury and on the procedures involved in neuropsychological testing (for notable exceptions including a group of brain-injured patients, see [27,28]). For most of the studied measures, naïve malingerers and malingerers who received information about the most common symptoms of brain injury (symptom coached simulators) were relatively easy to detect. In contrast, symptom-plus test-coached simulators (subjects receiving information about symptoms, a warning that neuropsychological testing includes tests designed to detect memory malingering, and some hints on how to perform on neuropsychological tests to avoid detection) were quite successful in passing the tests (for a recent review, see [29]). However, these studies only investigated measures derived from single tests [30].

### The present study

In the present study, the usefulness of the BSV-STM for the detection of memory malingering was investigated in an analog design using German participants. Furthermore, the sensitivity of measures derived from a standard test of memory function (VLMT) and of a test appearing more difficult than it actually is (FIT) were assessed for comparison purposes. Finally, the influence of coaching on test performance was explored. To this purpose, four groups of healthy subjects received different instructions one week prior to testing (best effort, BE; naïve simulators, NS; symptom coached simulators, SS; symptom plus test coached simulators, TS). Furthermore, a group of inpatients with mild to severe closed head injury performed the test with best effort.

## Methods

### Participants

123 undergraduate students or young professionals (n = 7; all holding a university degree) were randomly assigned to one of four groups (n = 33 best effort group; n = 29 naïve simulation group; n = 30 symptom coached group; n = 31 symptom plus test-coached group; for group description see below; see table 1 for demographic characteristics). All subjects were free of neurological diseases (past or present), had normal or corrected to normal visual acuity and were right handed. An additional group of 33 inpatients of a neurological rehabilitation clinic in Magdeburg performed the test with their best effort (PAT). Patients received rehabilitation after mild to severe closed head injury (mild: 3, moderate: 5, severe: 25; mean duration of coma: 27.3 days, range 0 to 240 days; mean duration of retrograde amnesia: 29.3 days, range 0 – 500 days; mean duration of anterograde amnesia: 10.7 days, range 0 to 45 days; for demographic information see table 1). Time from injury to assessment ranged from 1 month to 145 months (1 – 3 months: 10 patients, 4–12: 10, 12–24: 6, 24–48: 2, 48–120: 2, > 120: 3). None of the patients was currently involved in litigation. Patients were encouraged to perform the tests with their best effort and were assured that the results are only used for therapy planning in the clinic and, in an anonymous form, for a scientific study (no further explanation were given concerning the purpose of the study). The students received course credit for their participation whereas the young professionals were not compensated. Patients performed the tests as a part of routine neuropsychological assessment in the rehabiliatation clinic.

**Table 1:** Demographic characteristics of the sample

	**BE**	**NS**	**SS**	**TS**	**PAT**	
**Age**	22.60 (18–29)	22.50 (18–28)	22.70 (18–27)	21.80 (16–29)	35.30 (19–57)	*F*(4,151) = 29.70, *p *< .0001
**Sex**	12m 21w	11m 18w	11m 19w	11m 20w	23m 10w	*χ*^2 ^= 11.64, *p *< .05

ns = non-significant, alpha = .05. BE = best effort, NS = naïve simulators; SS = symptom coached group; TS = symptom plus test coached group, PAT = patient group.

Due to missing information on formal school education for the patients, we cannot provide statistical information on possible differences. However, all healthy participants had at least 13 years of schooling, whereas most of the patients had jobs requiring 10 years of schooling plus at least three years of additional vocational training. Thus, the patient group most likely had less school education compared to the healthy subjects.

The study protocol was approved by the ethical committee of Magdeburg University. All participants gave written informed consent prior to psychometric testing.

### Procedure

One week prior to testing all participants received a sealed envelope containing the instructions that differed according to group assignment. The best effort group (BE) was instructed to perform the given cognitive tests as best as they can. The naïve simulation group (NS) received the following scenario: "Imagine that you were involved in a car accident in which another driver hit your car. You were knocked unconscious and woke up in a hospital. You were kept overnight for observation and the doctors told you that you experienced a concussion. Imagine that after the accident, you are involved in a lawsuit against the driver of the other car. If you are found to have experienced significant injuries as a result of the accident, you are likely to receive a bigger settlement. You have decided to fake symptoms of a brain injury in order to increase the settlement you will receive. As part of the lawsuit, you are required to undergo cognitive testing to determine whether or not you have experienced a brain injury. If you can successfully convince the examiner that you have experienced significant brain damage, you are likely to get a better settlement. In the tests that you will have to undergo, I would like you to simulate brain damage, but in a believeable way, such that your examiner cannot tell that you are attempting to fake a brain injury" (presented in German; after [5]). The symptom coached group (SS) received additional information about the typical sequelae of brain injury (such as concentration and memory problems, headache, sleep disturbances etc). The symptom plus test coached group (TS) furthermore received the following information on how to approach testing:

- Tests that appear to be easy most likely are easy and can be solved by people with brain injuries.

- Performance of people with brain injury is consistent, i.e., try to perform equally well/equally bad in all tests that you will have to complete.

- Try not to perform too bad as most people with brain injury can at least answer some items in the tests that will follow.

Furthermore, participants were instructed not to talk to the examiner about their group assignment. After testing, the examiner debriefed the subject and the envelope containing the instructions was returned to the examiner. Furthermore, a postexperimental questionnaire was given to the subject asking how they approached the task and checking for compliance with the instructions. None of the subjects had forgotten the instructions given one week earlier.

The patient group was tested in the rehabilitation clinic with the instruction to perform the tests as best as they can.

### Cognitive Testing

The following cognitive tests were always administered in the same order:

- Aufmerksamkeits-Belastungstest d2 [31];

- VLMT [15];

- Subtests Alertness, divided attention and Go/NoGo from the „Testbatterie zur Aufmerksamkeitsprüfung" (TAP [32]);

- Rey 15 Items Test [14];

- Short Term Memory A from the Bremer Symptomvalidierung (STM-BSV; subtest of the TBFN) [13]);

- 8 Subtests from the Berliner Intelligenzstrukturtest (BIS [33]);

- Aufmerksamkeits-Belastungstest d2 [31].

The Test d2, the BIS, and the subtests of the TAP are commonly used standard neuropsychological tests in Germany. These were included to create a test situation that resembles standard cognitive testing. The results of these tests are not reported further in this paper.

### Test description and measures used to detect malingering

Only the three reported tests (VLMT, FIT, and Short-term memory form A from the BSV) are described in detail.

#### VLMT [15]

The VLMT is a German adaptation of the Rey Auditory Verbal Learning Test and consists of 15 words that are read one at a time by the examiner at a pace of one word per second. The examinee has to recall all the words that he can remember. This procedure is repeated five times. Then, a second list of 15 words is read and has to be recalled (interference list). In the 6^th ^trial, the original list must be recalled once again but is not read by the examiner. After 30 minutes, a delayed recall trial (trial 7) is performed followed by a recognition task. The recognition list contains the 15 original words, the 15 words from the interference list and 20 semantically or phonologically related new words.

The following measures were derived from the VLMT: supraspan (number of correctly recalled items in trial 1), number of recalled items in trials 5, total number of correctly recalled items in trials 1 to 5; number of correctly recalled items of the interference list; number of correctly recalled items in trial 6 (after the interference list), number of correctly recalled items after 30 minutes (delayed recall), loss due to interference (trial 6 – trial 5), loss due to forgetting over time (trial 7 – trial 5), number of correctly recognized words, corrected recognition (correctly recognized items – recognition errors), number of items at least three times recalled in trials 1 to 5 but not recognized, number of times the first word was recalled in trials 1 to 5, number of times the last word was recalled in trials 1 to 5.

#### Short-term memory form A from the BSV (STM-BSV)

This computerized test encompasses 100 trials consisting of two pictures each. The first picture contains a simple line drawing, whereas the second picture contains two complex line drawings. In one of these two pictures, the object shown on picture one is embedded. In a two-alternative-forced choice procedure, the participant has to decide which of the two drawings contains the object presented in picture one. Response times and accuracy are recorded. The test material consists of 20 different pictures that are presented in a randomized order. Each stimulus is repeated five times for a total of 100 trials. The following measures are analyzed: total correct responses and response time for correct responses.

#### Rey 15 Items Test

The FIT was performed according to standard instructions. Furthermore, we included the recognition trial developed by [14]. A sheet of paper containing 30 items (15 targets and 15 distracters that are similar to the original stimuli, e.g., the letter d) is given to the subject. The items that were presented in the learning phase have to be marked. The following indices were derived from the FIT: number of correctly recalled items, number of correctly recognized items, combination score: number of correctly recalled items + number of correctly recognized items.

### Data analysis

To determine which variables could discriminate between the five groups, we computed one-way ANOVAs with the factor GROUP (best effort, simulants, symptom coached, symptom + test coached, patients) and the Scheffé-contrasts for all variables.

Sensitivities and specificities were then computed at different cut-off values for all variables [34]. Cut-off scores were determined on the basis of patients' performance.

Parametric statistics were chosen for our analyses. As not all variables were distributed normally, we also conducted the respective nonparametric analysis. However, since all of the results of these two procedures were similar in magnitude and direction, we chose to report the results that we consider to be more user-friendly to clinicians, which are the parametric results.

## Results

### Group comparisons

Table 2 shows the means, standard errors and ANOVA-results for all variables. In general, the BE group performed best, followed by TS and PAT that both performed better than NS and SS. All one-way ANOVAs with the between subjects factor GROUP yielded a main effect of the GROUP-factor indicating that these variables could, in principle, discriminate between the different instruction conditions (see table 2).

**Table 2:** ANOVA-results and descriptive statistics

**Variable**	**Gr**	**Mean**	Standard deviation	**F(4,151)**	**P**	**Eta**
**BSV-STM correct responses (max. 100)**	BE	97.6	1.6	13.8	.0001	0.268
	NS	88.1	14.99			
	SS	85.1	10.48			
	TS	93.7	6.4			
	PA	97.8	3.04			
	T					
**BSV-STM RT correct responses**	BE	579 ms	120.1	4.14	.003	0.09
	NS	1130 ms	590.2			
	SS	1657 ms	1566.2			
	TS	1331 ms	1951.2			
	PA	870 ms	234.2			
	T					
**VLMT Trial 1**	BE	8.8	2.41	5.54	.0001	0.13
	NS	6.6	2.44			
	SS	6.7	2.62			
	TS	7.1	2.31			
	PA	6.1	2.57			
	T					
**VLMT Trial 5**	BE	13.4	1.66	10.18	.0001	0.21
	NS	10.3	2.9			
	SS	9.2	3.32			
	TS	11.2	2.75			
	PA	11.4	2.88			
	T					
**VLMT total Trials 1-5**	BE	59.1	8.95	9.7	.0001	0.21
	NS	43.8	12.02			
	SS	42.1	13.76			
	TS	49.4	12.66			
	PA	47.5	12.52			
	T					
**VLMT interference list**	BE	7.4	1.78	4.14	.003	0.1
	NS	5.6	1.68			
	SS	5.4	2.22			
	TS	5.8	2.39			
	PA	6	2.73			
	T					
**VLMT trial 6 (recall after interference)**	BE	12.7	2.17	11.97	.0001	0.24
	NS	8.2	4.17			
	SS	7.2	3.74			
	TS	8.8	3.37			
	PA	8.4	3.43			
	T					
**VLMT trial 7 (delayed recall)**	BE	12.7	1.99	12.31	.0001	0.25
	NS	7.7	4.5			
	SS	6.8	3.99			
	TS	8.1	3.6			
	PA	8.8	4.04			
	T					
**VLMT trial 6 – 5 (loss due to interference)**	BE	0.76	1.7	5.55	.0001	0.13
	NS	2.1	2.44			
	SS	1.97	1.61			
	TS	2.45	1.73			
	PA	2.88	2.1			
	T					
**VLMT trial 7 – 5 (loss due to delay)**	BE	0.7	1.61	6.4	.0001	0.15
	NS	2.62	2.8			
	SS	2.37	2.09			
	TS	3.33	2.04			
	PA	2.63	2.34			
	T					
**VLMT recognition**	BE	14.4	1.27	7.56	.0001	0.17
	NS	11.4	3.8			
	SS	10.7	3			
	TS	12.3	2.51			
	PA	12.3	2.99			
	T					
**VLMT corrected recognition score**	BE	14	1.72	16.56	.0001	0.31
	NS	8.1	5.91			
	SS	4.9	6.41			
	TS	9.6	4.55			
	PA	11.1	3.46			
	T					
**VLMT min 3 times recalled but not recognized**	BE	0.27	0.72	4.83	.001	0.11
	NS	1.24	1.41			
	SS	1.4	1.38			
	TS	0.84	1			
	PA	0.81	1			
	T					
**VLMT number of times the first word was recalled in trials 1 to 5**	BE	4.5	0.83	2.37	.055	0.06
	NS	3.7	1.58			
	SS	4.2	1.12			
	TS	4.3	1.08			
	PA	4.3	0.89			
	T					
**VLMT number of times the last word was recalled in trials 1 to 5**	BE	4.6	0.7	2.66	.035	0.07
	NS	4.1	1.03			
	SS	4	1.26			
	TS	4.1	1.15			
	PA	3.8	1.23			
	T					
**FIT recall**	BE	13.5	2.28	6.94	.0001	0.16
	NS	9.6	5.33			
	SS	9.4	4.17			
	TS	11.5	3.22			
	PA	9.4	4.16			
	T					
**FIT recognition**	BE	14.6	0.9	5.55	.0001	0.13
	NS	12.1	3.29			
	SS	11.9	2.38			
	TS	13.2	2.86			
	PA	13	2.55			
	T					
**FIT combination**	BE	28.1	2.56	7.88	.0001	0.17
	NS	21.7	7.67			
	SS	21	5.93			
	TS	24.4	5.64			
	PA	21.5	7.08			
	T					
**VLMT 1**	BE	39.4	5.2	16.3	.0001	0.3
	NS	24.1	13.6			
	SS	18.9	12.43			
	TS	27.3	9.76			
	PA	28.6	10.13			
	T					
**COMB 1**	BE	55	2.04	19.2	.0001	0.34
	NS	44.6	10.09			
	SS	41.7	7.85			
	TS	48.9	4.82			
	PA	49.3	5.17			
	T					

Eta = partial eta squared (effect size)

See text for explanation of variable abbreveations.

For most variables of the STM-BSV, VLMT and FIT, Scheffé-contrasts showed that (1) BE and PAT differed reliably from the simulation groups (exception: VLMT trial 6), (2) NS and SS groups performed worse than the TS group (exceptions: BSV-STM RT, VLMT trial 1, VLMT interference list, VLMT trial 6, VLMT trial 7, VLMT trial 6-5, VLMT trial 7-5), and (3) NS and SS groups did not differ (exception: VLMT corrected recognition). Thus, NS and SS groups were combined to form a new group of 59 subjects hereafter termed NSS (naïve and symptom coached simulators).

### Sensitivity of the tests to detect memory malingering

Tables 3 to 5 show the sensitivities and the specificity for each group and each variable of the VLMT, the FIT, and the STM-BSV. For these computations, data from the patient group was used to define the cut-off values. Thus, the column "specificity" lists the percentage of patients correctly classified as non-simulators and the percentage of subjects in the best effort group correctly classified as non-simulators at the respective cut-off value. The column "sensitivity" lists the percentage of subjects correctly classified as simulators in case of the NSS and TS groups. Overall, sensitivities were best for the STM-BSV-variables, whereas FIT and VLMT did not yield acceptable sensitivities.

**Table 3:** Sensitivity and specificity of the VLMT variables

**Variable**	**Cut-off**	**Specificity**		**Sensitivity**	
		**Patients**	**BE**	**NSS**	**TS**
**Trial 1**	0	100	100	0	0
	< 4	88	100	1.7	3.2
	< 5	79	97	17	9.7
	< 6	57	94	40.7	22.6
**Trial 5**	< 6	100	100	11.8	6.5
	< 8	88	100	23.7	6.5
	< 10	72	94	47.5	22.6
**Trial 1-5**	< 21	100	100	1.7	0
	< 31	90	100	22	6.5
	< 39	75	94	44	22.6
	< 43	57	94	47.5	29
**Interference list**	0	100	100	0	0
	< 4	90	100	10.2	9.7
	< 5	75	100	34	32.3
**Trial 6**	0	100	100	0	0
	< 5	90	100	25.4	9.7
	< 7	72	100	45.8	19.3
**Trial 7**	0	100	100	0	0
	< 2	90	100	3.9	3.2
	< 7	72	100	50.8	29
**Trial 6-5**	< -2	100	100	0	0
	< 1	90	100	18.6	9.7
	< 2	75	100	44	35.5
**Trial 7-5**	< -2	100	100	0	0
	< 0	90	100	13.6	0
	< 2	63	100	32.2	12.9
**Recognition**	< 4	100	100	3.9	0
	< 8	90	100	15.3	9.7
	< 12	75	97	50.8	25.8
**Corrected recognition score**	< 2	100	100	25.4	6.5
	< 6	90	100	40.7	6.5
	< 11	72	94	66.1	51.6
**3recallednotrecog**	> 4	100	100	5.1	0
	> 1	81	91	66.1	19.3
**VLMT number of times the first word was recalled in trials 1 to 5**	< 2	100	100	6.8	3.2
	< 4	78	84.9	25.4	16.1
	< 5	58	69.7	54.2	38.7
**VLMT number of times the last word was recalled in trials 1 to 5**	< 2	94	100	6.8	3.2
	< 3	82	97	10.2	6.5
	< 4	67	93.9	22	22.6

See text for an explanation of the variables and abbreviations.

**Table 4:** Sensitivity and specificity of the FIT variables

**Variable**	**Cut-off**	**Specificity**		**Sensitivity**	
		**Patients**	**BE**	**NSS**	**TS**
**FIT recall**	< 1	100	100	1.8	0
	< 4	82	100	20.3	0
	< 9	72	97	37.3	16.1
**FIT recognition**	< 6	100	100	1.8	6.5
	< 10	88	100	20.3	6.5
	< 12	78	97	42.4	16.1
**FIT combination score**	< 3	100	100	1.8	3.2
	< 15	88	100	17	6.5
	< 18	70	100	34	9.7
	< 22	61	97	47.5	19.4

See text for an explanation of the variables and abbreviations.

**Table 5:** Sensitivity and specificity of the STM-BSV variables

**Variable**	**Cut-off**	**Specificity**		**Sensitivity**	
		**Patients**	**BE**	**NSS**	**TS**
**BSV-STM correct responses**	< 84	100	100	28.8	9.7
	< 95	90	100	62.7	42
	< 98	73	54	88.1	67.7
**BSV-STM RT**	> 627	100	75	81.4	87.1
	> 633	90	75	79.7	83.9
	> 717	75	90	72.9	67.7
	> 774	60	90	71.2	54.8

See text for an explanation of the variables and abbreviations.

For the VLMT, at a specificity of 72–75%, the variables trial 7 (delayed recall), the corrected recognition score, the recognition score, and the total of trials 1 to 5 yielded the best classification rates (47.5% to 66.1% for the NSS group). For the FIT, the combination score (recall + recognition) provided the best results. For the STM-BSV, both variables (total correct responses, RT correct responses) yielded good sensitivities. For all variables, sensitivity for the NSS group was greater than for the TS group. Furthermore, the BE group participants were correctly categorized in at least 94% of cases by all variables.

The VLMT yields 11 scores. Only three of these scores showed sensitivities above 45% at a specificity above 70% for the simulator groups. We computed a combination score out of the three best VLMT-variables. This score (VLMT1 = VLMT trial 7 + VLMT trial 1-5 + VLMT corrected recognition score) classified 52.5% NSS and 26.7% TS participants correctly with a specificity of 75% (see table 6).

**Table 6:** Sensitivity and specificity of the combined scores

**Variable**	**Cut-off**	**Specificity**		**Sensitivity**	
		**Patients**	**BE**	**NSS**	**TS**
**VLMT 1**	< 24	100	100	0	0
	< 43	90	100	37.3	6.7
	< 57	75	100	52.5	26.7
**COMB 1**	< 43	100	100	15.3	0
	< 53	90	100	49.2	6.7
	< 58	75	100	57.6	20

VLMT1 = VLMT trial 7 + VLMT total + VLMT corrected recognition score; comb 1 = (VLMT1 + FIT comb + STM-BSV total correct responses)/3

VLMT-indices that require to keep track of previous responses (number of items at least three times recalled but not recognized) or knowledge about concepts of memory functioning (i.e., serial position effects; number of times the first and the last word are recalled in trials 1-5, respectively) were not superior to standard VLMT-variables in the detection of malingering.

Apart from the empirically derived cut-off values, the STM-BSV classifies the performance of the subjects based on the number of errors and on a probability analysis [13]. 29 of 33 BE participants and 29 of 33 non-litigating, non-simulating patients passed the test (corresponding to a specificity of 87.8%), while 40 of 59 NSS (corresponding to a sensitivity of 67.8%), and 14 of 31 TS (sensitivity 45.2%) participants failed the test. Using the cut-off scores derived from patient performance, 88.1% of the NSS and 67.7% of the TS group participants were correctly classified at a specificity of 73%.

To examine whether a combination score derived from the best measures of the three memory tests employed in the present study is useful for the detection of memory malingering, we developed the following combination score: comb1 = (VLMT1 + FIT comb + STM-BSV total correct responses)/3. At a specificity of 75%, this combination score classified 57.6% NSS, and 20% of the TS group participants correctly (see table 6).

Positive and negative predictive power (PPP and NPV, respectively) are diagnostic classification statistics that can be helpful in clinical decision making. PPP is the probability of the presence of a disorder (of malingering) in case of a positive test finding, NPP is the probability of the absence of a disorder (of malingering) given a negative test finding. Information on mathematical computing can be found in [34]. Table 7 shows NPP and PPP for the different cut-offs at a base-rate of 57.7% corresponding to the overall percentage of subjects instructed to simulate brain injury in the present study (90 of 156 participants).

**Table 7:** Negative (NPP) and positive predictive power (PPP) at the base-rate of .577 which corresponds to the 57.7% instructed simulators in the present experiment

**Variable**	**Cut-off**	**Specificity**	**Sensitivity**	**NPP**	**PPP**
**Trial 1**	< 4	94	2.2	.4	.33
	< 5	88	14.5	.33	.62
	< 6	75	34.5	.23	.65
**Trial 5**	< 6	100	10	.41	1
	< 8	94	17.7	.36	.8
	< 10	83	38.9	.26	.75
**Trial 1-5**	< 21	100	1.1	.43	1
	< 31	95	16.7	.37	.82
	< 39	85	36.6	.27	.76
	< 43	75	41.1	.22	.69
**Interference list**	< 4	95	10	.38	.73
	< 5	88	33.4	.29	.79
**Trial 6**	< 5	95	20	.36	.84
	< 7	86	36.7	.27	.78
**Trial 7**	< 2	95	3.7	.4	.5
	< 7	86	43.3	.26	.8
**Trial 6-5**	< 1	95	15.5	.37	.81
	< 2	88	41.1	.27	.82
**Trial 7-5**	< 0	95	8.9	.39	.70
	< 2	81	25.6	.27	.64
**Recognition**	< 4	100	2.6	.43	1
	< 8	95	13.4	.37	.78
	< 12	86	42.2	.26	.8
**Corrected recognition score**	< 2	100	18.9	.39	1
	< 6	95	28.9	.34	.89
	< 11	41	61.1	.07	.58
**3recallednotrecog**	> 4	100	3.3	.43	1
	> 1	86	50	.25	.83
**VLMT number of times the first word was recalled in trials 1 to 5**	< 2	100	5.5	.42	1
	< 4	82	22.2	.29	.62
	< 5	64	48.9	.16	.64
**VLMT number of times the last word was recalled in trials 1 to 5**	< 2	97	5.6	.41	.71
	< 3	90	8.9	.36	.54
	< 4	80	22.2	.28	.6
**FIT recall**	< 1	100	1.2	.43	1
	< 4	91	13.3	.35	.66
	< 9	85	30	.28	.73
**FIT recognition**	< 6	100	3.4	.43	1
	< 10	94	15.6	.36	.78
	< 12	88	33.3	.29	.79
**FIT combination score**	< 3	100	2.3	.43	1
	< 15	94	13.4	.37	.75
	< 18	85	25.7	.29	.70
	< 22	79	37.8	.24	.71
**BSV-STM correct responses**	< 84	100	22.2	.38	1
	< 95	95	55.6	.27	.94
	< 98	64	81.1	.11	.75
**BSV-STM RT**	> 627	88	83.4	.18	.9
	> 633	83	81.2	.17	.86
	> 717	82	71.1	.19	.84
	> 774	75	65.6	.17	.78
**VLMT 1**	< 43	95	26.8	.34	.88
	< 57	88	43.6	.27	.83
**COMB 1**	< 43	100	10	.41	1
	< 53	95	34.6	.32	.9
	< 58	88	44.7	.27	.83

Note: Sensitivity and specificity are computed from the combined values of the patients and best effort groups (specificity) and the combined values of all simulator groups (sensitivity).

NPP and PPP depend on the base-rate of the condition of interest [34]. Unfortunately, reliable data on the base rate of malingering of neurocognitive symptoms in Germany is not available. Given the differences in litigation legislature in Germany and in the U.S., we consider it inappropriate to rely on estimates originating in the U.S. Thus, table 8 shows NPP and PPP at three different base rates of malingering (10%, 20% and 30%). Please note that NPP and PPP are computed on the basis of sensitivities and specificities that include coached simulators. Thus, NPP and PPP reflect the fact that the tests used to detect malingering in the present study are all susceptible to coaching.

**Table 8:** Negative (NPP) and positive predictive power (PPP) at various base-rates of malingering

**Variable**	**Cut-off**	**Base rate**	**NPP**	**PPP**
**Trial 1**	< 6	.1	.50	.13
		.2	.44	.26
		.3	.38	.37
**Trial 5**	< 10	.1	.60	.20
		.2	.52	.36
		.3	.44	.50
**Trial 1-5**	< 43	.1	.50	.15
		.2	.43	.29
		.3	.37	.41
**Interference list**	< 5	.1	.68	.24
		.2	.59	.41
		.3	.50	.54
**Trial 6**	< 7	.1	.65	.23
		.2	.56	.40
		.3	.48	.53
**Trial 7**	< 7	.1	.64	.26
		.2	.55	.44
		.3	.46	.57
**Trial 6-5**	< 2	.1	.80	.26
		.2	.71	.44
		.3	.61	.57
**Trial 7-5**	< 2	.1	.59	.13
		.2	.52	.25
		.3	.45	.37
**Recognition**	< 12	.1	.64	.25
		.2	.55	.43
		.3	.47	.56
**Corrected recognition score**	< 11	.1	.15	.10
		.2	.13	.21
		.3	.12	.31
**3recallednotrecog**	> 1	.1	.64	.28
		.2	.54	.47
		.3	.45	.60
**VLMT number of times the first word was recalled in trials 1 to 5**	< 5	.1	.36	.13
		.2	.31	.25
		.3	.27	.37
**VLMT number of times the last word was recalled in trials 1 to 5**	< 4	.1	.57	.11
		.2	.51	.22
		.3	.44	.32
**FIT recall**	< 9	.1	.64	.18
		.2	.56	.33
		.3	.48	.46
**FIT recognition**	< 12	.1	.68	.24
		.2	.59	.41
		.3	.50	.54
**FIT combination score**	< 22	.1	.55	.17
		.2	.48	.31
		.3	.41	.44
**BSV-STM correct responses**	< 84	.1	.34	.20
		.2	.28	.36
		.3	.23	.49
**BSV-STM RT**	> 774	.1	.48	.23
		.2	.40	.40
		.3	.33	.53
**VLMT 1**	< 57	.1	.67	.29
		.2	.58	.48
		.3	.48	.61
**COMB 1**	< 58	.1	.67	.29
		.2	.58	.48
		.3	.48	.61

## Discussion

The present study examined the usefulness of the BSV-STM for the detection of feigning memory impairments. Furthermore, the influence of different coaching methods on the accuracy of simulation detection was investigated in an analog design. Four groups of neurologically healthy participants and a group of brain-injured inpatients of a neurological rehabilitation clinic were given the VLMT, the FIT, and the STM-BSV as part of a larger neuropsychological test battery. To reiterate, besides a best effort group, three simulator groups with different levels of prior information (naïve, symptom coached, symptom plus test coached, NS, SS, TS, respectively) were created. Overall, the NS and the SS were the easiest to detect and did not differ in performance, whereas the TS group was the hardest to detect. The scores derived from the used symptom-validity test, the STM-BSV, showed the best sensitivity but was sensitive to coaching. A standard neuropsychological test, the VLMT, and the FIT as well as the combination scores derived from several tests failed to provide acceptable sensitivities.

This is the first study investigating the usefulness of the STM-BSV for detection of incomplete effort in an analog design. This symptom validity test yielded a satisfactory specificity (all participants of the best effort group passed the test). However, the BSV-STM was sensitive to coaching: at a cut-off of < 98 correct responses (corresponding to 73% specificity) 88.1% of naïve and symptom coached simulators were detected. In contrast, only 67.7% of a group receiving additional information on how to approach effort testing were categorized correctly. Thus, one has to be aware that the clinical usefulness of the STM-BSV in detecting memory malingering can be diminished if the subject is informed how to approach effort testing.

All other measures used to detect memory malingering were also sensitive to coaching, and this is especially true for the symptom plus test coached group. Thus, it seems that irrespective of the method used, sophistically coached memory malingerers are hard to detect (see also [29]).

In clinical practice, malingering tests should have a specificity of at least 90%. Note that in the current study, cut-offs were derived from the performance of patients with mostly severe closed head injuries. Thus, the cut-offs derived from these data can be seen as quite conservative and it might be acceptable to lower the specificity required under such conditions. However, the main findings of the present study also hold at a specificity of 90%: (1) the BSV-STM yields the highest sensitivities but is sensitive to coaching (sensitivities: BE: 100, NSS: 62.7, TS: 42 for number of correct responses); (2) the FIT (max. sensitivity for the simulation groups 20.3) and the VLMT (max. sensitivity for the simulation groups 40.3) do not yield satisfactory sensitivities and (3) the combination scores cannot improve the situation substantially (max. sensitivity for the simulation groups 49.2).

Previous studies using the FIT and the VLMT for malingering detection have yielded mixed results, but most are generally in line with our observation of sensitivities that are too low for clinical use in memory malingering detection. For example, using the FIT, Boone and coworkers also reported rather low sensitivities at a specificity above 85% ranging from 5% to 86% (for references, see tables 1 and 2 in [14]). Given these findings, several attempts have been made to improve the FIT. Boone and colleagues [14] introduced the recognition procedure and showed that a cut-off value of < 20 of a combination score (sum of correctly reproduced and correctly recognized items) improved sensitivity to 71% (at >= 92% specificity). However, for the current data set, this combination score did not enhance sensitivity compared to the recall score (cut-off < 22: from 37.3% to 34% in the present study for the NSS group; from 16.1% to 9.7% at 70% specificity for the TS group; from 59.2% to 71% at 95% specificity in Boone et al. [14]). Thus, we could not replicate the usefulness of the addition of the recognition trial.

To our knowledge, this is the first study of simulated memory impairment detection using the VLMT. However, as the VLMT is the German adaptation of the AVLT, it might be possible to compare these two tests. In the present study, delayed recall, the sum of words recalled in trials 1 to 5, and the corrected recognition score yielded the best classification rates (45.8% to 66.1% for the NSS group, 29% to 51.6% for the TS group at 75% specificity). For comparison, [16] report sensitivities of 40.4% (trial 7) and 21.3% (corrected recognition) in a sample of real world suspected malingerers at above 90% specificity. Thus, we obtained grossly comparable sensitivities albeit at a lower specificity that is most likely caused by using a sample of mostly severe head injured patients for deriving the cut-off scores in the present investigation.

In contrast to previous work [35], the presence of primacy- and recency-effects could only detect the most "severe" cases of memory malingering in our study. We tried several operationalizations of the presence of serial position effects (sum of words 1–5, 6–10, 11–15 recalled in trials 1-5, serial position effect present if both, recall of words 1–5 and 11–15 is larger than recall of words 6–10; number of times the first word is recalled in trails 1-5; number of times the last word is recalled in trails 1-5) none of which yielded satisfactory sensitivities. Most patients with moderate to severe head injury were able to recall the first and the last word of the list at least 4 times, but most of the subjects instructed to malinger memory impairment also did. Furthermore, words that were recalled at least three times in trial 1-5 but not recalled have been proposed as an index of memory malingering (termed index 1 by [36]). In the present study, this index was slightly superior to the best standard indices of the VLMT indicating that more complex measures requiring to keep track of previous memory performance are good candidates for the detection of feigned memory impairment. Furthermore, the inclusion of a second delayed recognition trial as proposed by Barrash and colleagues [26] in the extended AVLT might improve the usefulness of the VLMT in the detection of memory feigning.

The NPP and PPP values shown in tables 7 and 8 can be used to assess the usefulness of the different variables in clinical decision making. Note that these tables are computed on the basis of sensitivities and specificities derived from the complete sample, i.e. including the coached simulators. Thus, the values reflect the difficulties of the tests to detect coached malingerers. We think that computing NPP and PPP in this way (and not separately for each group) is more appropriate to the situation of the clinician who does not know whether an examinee was coached prior to the assessment session.

### Methodological limitations of the study

Several methodological limitations of this study have to be considered. First, the present findings are limited by the use of a simulated malingering design. More specifically, the use of simulators might decrease generalizability of the results. However, some evidence in support of the simulation design has been presented showing that student malingerers perform similar to mild traumatic brain injury patients [37,38]. In the present study, however, patients performed better in most tests compared to the student simulators. Furthermore, the use of student populations who have no financial incentive to simulate malingering may also limit generalizability. Research shows that financial compensation does affect patients' performance in clinical contexts [39,40]. Thus, most likely the absence of significant financial incentives for the participants in the present study influenced their performance. Furthermore, it has to be considered that a sample of university students with above-average intelligence has been employed. Thus, it is an open issue whether the same results would be obtained with a sample of simulators of average or below-average IQ.

The cut-offs used in the present study to compute the sensitivity and the specificity of the tests for the detection of memory malingering were derived from a sample of non-litigating patients with mild to severe closed head injury that were instructed to perform the neurocognitive assessment with their best effort. It has been shown that a considerable percentage of patients in such heterogenous samples perform below the cut-off suggested by the test developers [41]. Thus, our use of cut-offs derived from such a sample can be considered as conservative. Moreover, it increases the clinical utility of the findings.

## Conclusion

The present analog study is the first to document the usefulness of the STM-BSV as a test of memory malingering. However, clinicians have to be aware that the BSV-STM is sensitive to coaching. Furthermore, we showed that the FIT and the VLMT are not clinically useful for the detection of memory malingering when cut-offs derived from real-world, mild to severely head injured patients are used.

## Abbreviations

ANOVA: Analysis of Variance; AVLT: Rey's Auditory Verbal Learning Test; BE: best effort group; BIS: Berliner Intelligenz-Strukturtest; FIT: Rey's 15 Item Test; NPP: Negative Predictive Power; NS: naïve simulators group; NSS: ns and ss groups combined; PAT: patient group; PPP: Positive Predictive Power; SS: symptom coached simulators group; STM-BSV: Short-term memory test, form A of the Bremer Symptom Validierung; TAP: Testbatterie zur Aufmerksamkeitsprüfung; TBFN: Testbatterie zur forensischen Neuropsychologie; TOMM: Test of Memory Malingering; TS: symptom plus test coached simulator group; VLMT: Verbaler Lern- und Merkfähigkeitstest.

## Competing interests

The authors declare that they have no competing interests.

## Authors' contributions

JR designed the study and wrote the manuscript. AB and UK collected the data. JR and AB analyzed the data. AB, MS and TFM helped substantially in interpreting the data.

## Pre-publication history

The pre-publication history for this paper can be accessed here:



## References

[B1] Heilbrun K, Bennett WS, White AJ, Kelly J (1990). An MMPI-based empirical model of malingering and deception. Behavioral Sciences and the Law.

[B2] Youngjohn JR, Burrows L, Erdal K (1995). Brain damage or compensational neurosis? The controversial post-concussive syndrome. Clin Neuropsychol.

[B3] Gouvier WD, Prestholdt PH, Warner MS (1988). A survey of common misconceptions about head injury and recovery. Arch Clin Neuropsychol.

[B4] Mittenberg W, Azrin R, Millsaps C, Heilbronner R (1993). Identification of malingered head injury on the Wechsler Memory Scale Revised. Psychol Assessment.

[B5] Suhr JA, Gunstad J, Greub B, Barrash J (2004). Exaggeration index for an expanded version of the Auditory Verbal Learning Test: Robustness to coaching. J Clin Exp Neuropsychol.

[B6] Vakil E (2005). The effect of moderate to severe traumatic brain injury (TBI) on different aspects of memory: a selective review. J Clin Exp Neuropsychol.

[B7] Langeluddecke PM, Lucas SK (2005). WMS-III findings in litigants folloowing moderate to extremely severe brain trauma. J Clin Exp Neuropsychol.

[B8] Heilbronner RL (2004). A status report on the practice of forensic neuropsychology. Clin Neuropsychol.

[B9] Rabin LA, Barr WB, Burton LA (2005). Assessment practices of clinical neuropsychologists in the United States and Canada: a survey of INS, NAN, and APA division 40 members. Arch Clin Neuropsychol.

[B10] Slick DJ, Tan JE, Strauss EH, Hultsch DF (2004). Detecting malingering: a survey of experts' practices. Arch Clin Neuropsychol.

[B11] Schagen S, Schmand B, de Sterke S, Lindeboom J (1997). Amsterdam Short-Term Memory Test: A new procedure for the detection of feigned memory deficits. J Clin Exp Neuropsychol.

[B12] Green P (2003). Green's Word Memory Test. User's manual.

[B13] Heubrock D, Petermann F (2000). Testbatterie zur forensischen Neuropsychologie (TBFN).

[B14] Boone KB, Salazar X, Lu P, Warner-Chacon K, Razani J (2002). The Rey 15 Item recognition trial: a technique to enhance sensitivity of the Rey 15-Item Memorization test. J Clin Exp Neuropsychol.

[B15] Helmstaedter C, Lendt M, Lux S (2001). Verbaler Lern- und Merkfähigkeitstest (VLMT).

[B16] Larrabee GJ, Larrabee GJ (2007). Identification of malingering by pattern analysis on neuropsychological tests. Assessment of malingered neuropsychological deficits.

[B17] Boone KB, Lu PH, Larrabee GJ (2007). Non-forced-choice effort measures. Assessment of malingered neuropsychological deficits.

[B18] Lynch WJ (2004). Determination of effort level, exaggeration, and malingering in neurocognitive assessment. J Head Trauma Rehabil.

[B19] Vickery CD, Berry DT, Inman TH, Harris MJ, Orey SA (2001). Detection of inadequate effort on neuropsychological testing: a meta-analytic review of selected procedures. Arch Clin Neuropsychol.

[B20] Tombaugh TN (1996). Test of Memory Malingering (TOMM).

[B21] Binder LM (1993). Assessment of malingering after mild head trauma with the Postland Digit Recognition Test. J Clin Exp Neuropsychol.

[B22] Wetter MW, Corrigan SK (1995). Providing information to clients about psychological tests: a survey of attorneys' and law students' attitudes. Professional Psychology: Research and Practice.

[B23] Essig SM, Mittenberg W, Peterson RS, Strauman S, Cooper JT (2001). Practices in forensic neuropsychology: perspectives of neuropsychologists and trial attorneys. Arch Clin Neuropsychol.

[B24] Bauer L, McCaffrey RL (2006). Coverage of the Test of Memory Malingering, Victoria Symptom Validity Test, and Word Memory Test on the internet: is test security threatened. Arch Clin Neuropsychol.

[B25] Suhr JA, Barrash J, Larrabee GJ (2007). Performance on standard attention, memory, and psychomotor speed tasks as indicators of malingering. Assessment of malingered neuropsychological deficits.

[B26] Barrash J, Suhr J, Manzel K (2004). Detecting poor effort and malingering with an expanded version of the Auditory Verbal Learning Test (AVLTX): Validation with clinical samples. J Clin Exp Neuropsychol.

[B27] Glassmire DM, Bierley RA, Wisniewski AM, Greene RL, Kennedy JE, Date E (2003). Using the WMS-III faces subtest to detect malingered memory impairment. J Clin Exp Neuropsychol.

[B28] Hilsabeck RC, LeCompte DC, Marks AR, Grafman J (2001). The Word Completion Memory Test (WCMT): a new test to detect malingered memory deficits. Arch Clin Neuropsychol.

[B29] Suhr JA, Gunstad J, Larrabee GJ (2007). Coaching and malingering: a review. Assessment of malingered neuropsychological deficits.

[B30] Merten T, Green P, Henry M, Blaskewitz N, Brockhaus R (2005). Analog validation of German. language symptom validity tests and the influence of coaching. Arch Clin Neuropsychol.

[B31] Brickenkamp R (2002). Test d2.

[B32] Zimmermann P, Fimm B (2002). Testbatterie zur Aufmerksamkeitsprüfung (TAP). Version 1.7.

[B33] Jäger AO, Süß HM, Beauducel A (1997). Berliner Intelligenzstruktur-Test (Form 4, BIS-4).

[B34] Larrabee GJ, Berry DTR, Larrabee GJ (2007). Diagnostic classification statistics and diagnostic validity of malingering assessment. Assessment of malingered neuropsychological deficits.

[B35] Powell MR, Gfeller JD, Hendricks BL, Sharland M (2004). Detecting symptom- and test-coached simulators with the test of memory malingering. Arch Clin Neuropsychol.

[B36] Suhr JA, Tranel D, Wefel J, Barrash J (1997). Memory performance after head injury: contributions of malingering, litigation status, psychological factors, and medication use. J Clin Exp Neuropsychol.

[B37] Brennan AM, Gouvier WD (2006). Are we honestly studying malingering? A profile and comparison of simulated and suspected malingerers. Applied Neuropsychol.

[B38] Haines ME, Norris MP (2001). Comparing student and patient simulated malingerers' performance on standard neuropsychological measures to detect feigned cognitive deficits. Clin Neuropsychol.

[B39] Binder LM, Rohling ML (1996). Money matters: meta-analytic review of the effects of financial incentives on recovery after closed-head injury. Am J Psychiat.

[B40] Binder LM, Willis SC (1991). Assessment of motivation after financially compensable minor head trauma. Psychol Assessment.

[B41] Merten T, Bossink L, Schmand B (2007). On the limits of effort testing: symptom validity tests and severity of neurocognitive symptoms in nonlitigating patients. J Clin Exp Neuropsychol.

